# The cleaved FAS ligand activates the Na^+^/H^+^ exchanger NHE1 through Akt/ROCK1 to stimulate cell motility

**DOI:** 10.1038/srep28008

**Published:** 2016-06-15

**Authors:** Michael Monet, Mallorie Poët, Sébastien Tauzin, Amélie Fouqué, Auréa Cophignon, Dominique Lagadic-Gossmann, Pierre Vacher, Patrick Legembre, Laurent Counillon

**Affiliations:** 1 Université Nice-Sophia Antipolis, CNRS UMR7370 LP2M, Faculté de Médecine, 28 Avenue de Valombrose 06107 Nice cedex. France and Laboratories of Excellence Ion Channel Science and Therapeutics, France; 2 Université Rennes-1, Inserm U1085, 2 Av du Prof. Léon Bernard, 35043, Rennes, France; 3 Centre Eugène Marquis, Inserm ER440, Oncogenesis Stress and Signaling, Equipe Labellisée Ligue Contre Le Cancer “Death Receptors and Tumor Escape”, rue bataille Flandres-Dunkerque, 35042 Rennes, France; 4 Université de Bordeaux, Inserm U1045, 146 rue Léo Saignat, 33046 Bordeaux, France; 5 Bergonié Cancer Institute, 33000 Bordeaux, France

## Abstract

Transmembrane CD95L (Fas ligand) can be cleaved to release a promigratory soluble ligand, cl-CD95L, which can contribute to chronic inflammation and cancer cell dissemination. The motility signaling pathway elicited by cl-CD95L remains poorly defined. Here, we show that in the presence of cl-CD95L, CD95 activates the Akt and RhoA signaling pathways, which together orchestrate an allosteric activation of the Na^+^/H^+^ exchanger NHE1. Pharmacologic inhibition of Akt or ROCK1 independently blocks the cl-CD95L-induced migration. Confirming these pharmacologic data, disruption of the Akt and ROCK1 phosphorylation sites on NHE1 decreases cell migration in cells exposed to cl-CD95L. Together, these findings demonstrate that NHE1 is a novel molecular actor in the CD95 signaling pathway that drives the cl-CD95L-induced cell migration through both the Akt and RhoA signaling pathways.

The transmembrane form of CD95L (FasL) interacts with the CD95 death receptor (Fas receptor) to induce the formation of the CD95/FADD/Caspase-8/10 complex (also called Death Inducing Signaling Complex, DISC) and, consequently, apoptosis^1^. This process, which involves the death domain (DD) of the ligand, has been widely documented and is considered crucial for immune system maintenance and for the ligand’s anti-tumorigenic effects^1^. However, a distinct and less well understood aspect of CD95L is that it can also be cleaved by metalloproteases and released into the bloodstream as a soluble, homo-trimeric ligand (referred to as cl-CD95L); this cleaved ligand fails to induce the formation of the DISC, and has been traditionally considered either as an inactive form or as a competitive inhibitor of the apoptotic transmembrane ligand^2^. Recent studies, however, have raised other possibilities regarding the potential function of the cleaved form of the ligand. For example, we recently reported that, in lymphocytes, the interaction of cl-CD95L with CD95 leads to cortical actin reorganization, increased intracellular calcium levels, and activation of the phosphoinositide 3-kinase (PI3K)/Akt signaling pathway, and that these events are correlated with accelerated cell migration^3^. Further, the cleaved ligand is found in high concentrations in the serum of Systemic Lupus Erythematosus (SLE), where it contributes to the pathology of chronic inflammatory disorders via a molecular mechanism that remains to be identified^3^,^4^. In addition, cl-CD95 has been shown to exert pro-oncogenic functions by promoting the survival of ovarian and liver cancers^5^, the invasion of glioblastomas^6^ and of triple-negative breast cancer cells^7^, and resistance to chemotherapy in lung cancers^8^. In view of these disparate cellular responses produced by the same ligand, as well as their various pathological manifestations, we wanted to investigate the downstream targets of cl-CD95L.

The Na^+^/H^+^ exchanger NHE1 appeared to be one promising candidate. NHE1 is a ubiquitous plasma membrane transporter whose main function is to regulate intracellular pH and cell volume (for review see^9^). NHE1 can participate in cell spreading and migration^10^,^11^, by serving as a cytoskeletal anchoring platform and acting to maintain an asymmetrical gradient of protons along the axis of cell migration^12^. In addition, NHE1-driven modulation of pH contributes to structural changes in proteins such as cofilin^13^, and NHE1 has been reported to play an active role in invadopodia, structures involved in tumor cell metastasis^14^,^15^. Finally, in previous studies, we had shown that cl-CD95 can lead to the activation of Akt^3^ and to elevated intracellular calcium levels, both of which are known to activate NHE1^16^,^17^.

The present study shows for the first time that cl-CD95 activates NHE1, which thereby enhances cell migration. Using a combination of inhibitors and site-directed mutagenesis, we subsequently demonstrate that this mechanism occurs through a modification of NHE1 cooperative response to intracellular protons and that it involves both the Akt and RhoA-dependent pathways.

## Results and Discussion

### cl-CD95L induces cell migration through an NHE1-driven mechanism

To investigate the link between CD95 and NHE1, we stably transfected the fibroblast cell line PS120, which lacks both CD95 and NHE1^18^, with either a wild type (WT) or DD-truncated CD95 (CD95^(1–210)^) (Fig. S1A) together with WT or transport inactive NHE1 (D267V mutant, see further in the text). We first validated this cell model by showing that a previously described multi-aggregated form of CD95L (Ig-CD95L) consisting of the extracellular region fused to a dimerization Ig domain derived from the LIF receptor gp190 19 could fully induce the apoptotic signal in the presence of wild type CD95 (Fig. S1B); in the presence of the CD95^(1–210)^ death-domain deficient CD95 mutant, in contrast, cell death did not occur.

**Figure 1:**
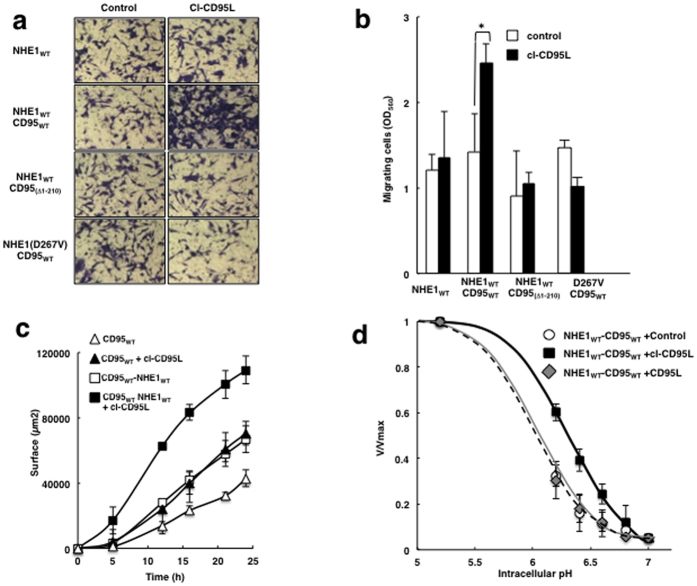
cl-CD95L increases cell migration by activation of NHE1. (**a**) PS120 cells, stably expressing different combinations of wild type or transport inactive mutant of NHE1 (D267V), and wild type or death domain mutant of CD95, were incubated for 16 h in Boyden chambers in the presence or absence of 100 ng/ml of cl-CD95 in the lower compartment. Migrating cells were then fixed with methanol and stained by Giemsa. For each experiment, five pictures of random fields were taken and a representative picture is shown (Bars = 70 μm).(**b**) For quantification, Giemsa-stained migrating cells were lysed, and absorbance was measured at 560 nm. Values represent means and SD of three independently performed experiments. (**c**) Cell migration of NHE1 deficient PS120-CD95 cells (▴), or PS120-CD95 cells stably expressing wild type NHE1 (◾) was tested using wound-healing assays in the presence (full symbols) or absence (empty symbols) of cl-CD95L (100 ng/ml). For this, straight scratches of identical size were executed on confluent cell monolayers preincubated in 0.5% Fetal Calf Serum. Medium containing 100 ng/ml of cl-CD95 ligand then was added and images were acquired for 24 hours using phase contrast microscopy. Quantification was performed using ImageJ (see methods). For each condition, images were acquired at five different positions along each scratch. The graphs are representative of 3 independent experiments. The corresponding images are displayed in Supplementary Figures. (**d**) NHE1 cooperative activation by intracellular protons was measured in PS120-NHE1-CD95 cells treated for 10 minutes with 100 ng/ml of cl-CD95L, CD95L or control medium. Intracellular pH was clamped at different pH values as described in Materials and Methods. Initial rates of NHE1 activity were measured using rapid kinetics of Li^+^ uptake and are represented as normalized values to the maximal uptake (V/Vmax). Each line represents the activity of NHE1 in one of the experimental conditions. ♦-Grey Line: CD95 ligand, ○-dotted line: untreated; ◾ dark line: cl-CD95 ligand. Data are representative for at least five independent experiments. Error bars are SEM.

When we exposed these cells to cl-CD95L and observed them using Boyden chamber and wound healing assays to measure cell motility, we found that the ligand induced migration in these cells when wild type CD95 was expressed (hereafter referred to as PS120-NHE1-CD95 cells; Fig. 1a–c and S1C); the DD-deficient form of CD95 (CD95^(1–210)^), in contrast, did not allow motility. These results indicated that the PS120 cell line is an exceptional tool for studying the downstream effectors of CD95 signaling, including Na^+^/H^+^ exchange, as it act as a recipient that can be reconstituted with any combination of WT and/or mutated forms of CD95 as well as Na^+^/H^+^ exchangers.

We next investigated whether NHE1 was a target of cl-CD95. To do this, we used an assay based on certain kinetic features of NHE1, notably the fact that its transport rate is virtually zero at physiological pH but increases in a sigmoidal manner when the intracellular pH drops^20^,^21^. This particular behavior is the hallmark of a cooperative mechanism, and shapes the ability of NHE1 to maintain a steady-state pH^22^. Significantly, factors that modulate NHE1, such as hormonal or growth factor signals^9^, or changes in membrane shape and tension, modify its activity by detectably shifting the slope of the sigmoidal dose-response curve to intracellular protons^21^,^23^. It is known that, at a molecular level, this effect is mediated by the large intracellular C-terminal region of NHE1, which contains multiple phosphorylation sites and interaction sites for lipids and signaling proteins (for review see^24^). For our purposes, we were thus able to test the potential effects of cl-CD95L (non-apoptotic ligand) and Ig-CD95L (apoptotic ligand) on NHE1 activity by measuring NHE1’s dose response to various concentrations of intracellular protons in the presence or absence of these ligands.

As shown in Fig. 1d, stimulating PS120-NHE1-CD95 cells with cl-CD95L produced a marked shift of the sigmoidal dose response curve of NHE1 towards more alkaline intracellular pH, showing unambiguously that NHE1 became more sensitive to cytosolic protons in response to cl-CD95L acting on its canonical receptor, CD95. To our knowledge, this was the first demonstration of a direct functional link between cl-CD95L stimulation and NHE1 activation.

As NHE1 activation occurs with many stimuli that do not trigger motility, the next step was to investigate whether NHE1 was involved in the CD95-driven cell motility. For this purpose, we expressed the NHE1 mutant D267V in PS120 CD95 cells. This mutation yielded a transport-dead NHE1 molecule that was processed in a similar way to WT-NHE1 (Fig. S2A) but that was unable to mediate Na^+^/H^+^ exchange (Fig. S2B,C). Compared to WT-NHE, the expression of the mutant failed to implement the CD95-mediated cell motility in the Boyden chamber (Fig. 1a,b) and wound healing assays (Fig. S1D). These findings demonstrated that the CD95-mediated activation of functional NHE1 contributes to the cell migration observed upon stimulation with cl-CD95.

### The CD95-mediated activation of NHE1 occurs through an Akt-mediated mechanism

**Figure 2:**
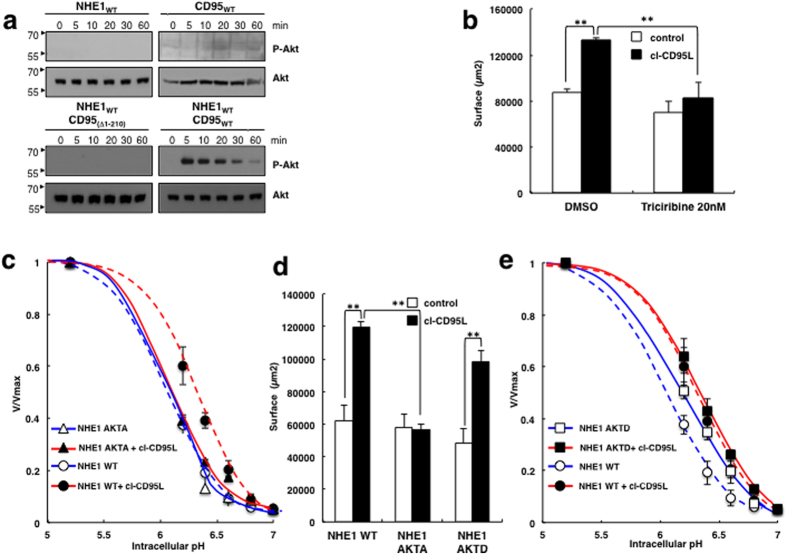
CD95-mediate Akt stimulation and activates NHE1. (**a**) After treatment with 100 ng/ml of cl-CD95L, cells were lysed and the levels of phosphorylated Akt (Ser 473) and total Akt were analyzed by immunoblotting using anti-Akt and anti-phosphoS473 Akt antibodies. Loading control: Total Akt. The analyzed cell lines are indicated on top of each Western Blot for p-Akt and total Akt. (**b**) Confluent cell monolayers (PS120-CD95-NHE1) were pre-incubated in presence or absence (noted DMSO) of the Triciribine (20 nM) for 1 hour and then treated as in Fig. 1c, in the presence or absence of cl-CD95L (100 ng/ml) for wound-healing assay. Histogram bars correspond to the surface covered after 24 hours. Error Bars are SEM, p < 0.05 (*), p < 0.01 (**). (**c**) The dose response of the PS120-CD95-NHE1 AKTA mutant (full lines, ▴) for intracellular protons was measured by Lithium uptake at clamped intracellular pH values in the presence (red line) or absence of cl-CD95L (100 ng/ml) (blue line) as described in Fig. 1d and compared to that the wild type of NHE1 response, (PS120-CD95-NHE1 WT dotted lines, ⦁) in the presence (red line) or absence of 100 ng/ml cl-CD95L (10 ng/ml) (blue line). Initial rates of NHE1 are represented normalized values to the maximal uptake (V/Vmax). Data are representative for at least three independent experiments. Error bars are SEM. (**d**) Cell migration of PS120-CD95 cells stably expressing wild type or AKTA or AKTD NHE1 mutants was assessed by wound healing assays. Histogram bars correspond to the surface covered after 24 hours. Error bars are SEMs. p < 0.05 (*), p < 0.01 (**). (**e**) The dose response of the PS120-CD95-NHE1 AKTD mutant (full lines, ◾) to intracellular protons was measured by lithium uptake in the presence (red line) or absence of 100 ng/ml cl-CD95L (blue line) as described in Fig. 1d and compared to that of wild type NHE1 response (PS120-CD95-NHE1 WT dotted red and blue lines, ⦁). Initial rates of NHE1 are represented normalized values to the maximal uptake (V/Vmax). Data are representative for at least three independent experiments. Error bars are SEM.

These results prompted us to investigate the molecular mechanism by which CD95 stimulation by the cleaved ligand activates Na^+^/H^+^ exchange. We had previously shown that cl-CD95L induces the phosphorylation of Akt at serine 473, a hallmark of PI3K activation in lymphocytes^3^. We found that the same signaling event was triggered in PS120-NHE1-CD95 cells (Fig. 2a). By contrast, in fibroblasts devoid of CD95 or expressing a DD-deficient CD95, the naturally processed ligand did not induce Akt phosphorylation. Strikingly, cells expressing a fully functional CD95 receptor but lacking expression of NHE1 failed to implement serine 473 phosphorylation in presence of cl-CD95L. This points towards an important role of NHE1 in Akt activation. As NHE1 possesses a long C-terminal tail that binds a large set of signaling molecules, these data indicate that this region may serve as a scaffold for the complete Akt activation in CD95-stimulated cells.

Triciribine (20 nM), a selective and reversible Akt inhibitor^25^, prevented cell motility in PS120-NHE1-CD95 cells exposed to cl-CD95L (Figs 2b and S2D), confirming that the activation of Akt is instrumental in cl-CD95-mediated cell migration.

As Akt is known to activate NHE1 at its serine 648^16^,^26^, we next generated a S648A NHE1 mutant (AKTA mutant) in the C-terminal tail of NHE1. The AKTA mutant was fully able to exchange Na^+^ and H^+^ and could still be activated by 20% Fetal Calf Serum, indicating that this mutation preserved the ability of NHE1 to transport and to be regulated (Fig. S2E). However, this mutant was unable to be allosterically-activated by cl-CD95L (Fig. 2c), as its dose response curve to intracellular protons was not shifted by the cleaved ligand. Also, in contrast to WT-NHE1, the AKTA mutant did not enhance CD95-mediated cell migration when expressed in PS120-CD95 cells (Fig. 2d). Together, these results supported that NHE1 responds through its Serine 648 to the Akt pathway following its stimulation by cl-CD95L.

We next aimed to mimic the phosphorylation of this crucial site by constructing a S648D (AKTD) phosphomimetic mutant. Similar to AKTA, this mutant was functional and showed a cooperative response to intracellular protons, which could be enhanced by serum (Fig. S3A). This mutant displayed an interesting phenotype. As shown in Fig. 2e, the AKTD dose response curve for protons was noticeably shifted to the right, even in the absence of ligand, indicating that the phosphomimetic mutation produced a slight constitutive-active phenotype. However, the addition of the cl-CD95L shifted the curve even further (Fig. 2e), suggesting that Akt is not the only pathway through which cl-CD95L activates NHE1. Consistently, the AKTD mutant did not by itself display any accelerated migration in the absence of ligand; this was only achieved with exposure of the PS120-CD95 AKTD cells to the cleaved ligand (Fig. 2d and Fig. S2D). The fact that the Akt-mediated activation of NHE1 was not sufficient to enhance migration by itself is not surprising, as cell migration is a complex process that requires multiple events besides the activation of Na^+^/H^+^ exchange. Taken together, these results suggested that the Akt-mediated activation of NHE1 is necessary but not sufficient for full activation of NHE1 and, as a result, of migration, and that at least one additional event is also required.

### NHE1 activity is stimulated by the serine/threonine kinase ROCK upon CD95 engagement

We recently showed that CD95 induces a Ca^2+^ response in activated T lymphocytes and breast cancer cells. Because NHE1 can be activated by intracellular calcium^17^, we next investigated whether this increase in calcium could be a second event that is essential for full NHE1 activation in cells exposed to cl-CD95L. As shown in Fig. S3B, no Ca^2+^ signal was detected in NHE1-CD95-PS120 cells exposed to cl-CD95L, indicating that, in this cell line, intracellular calcium does not contribute to the CD95-mediated activation of NHE1.

**Figure 3:**
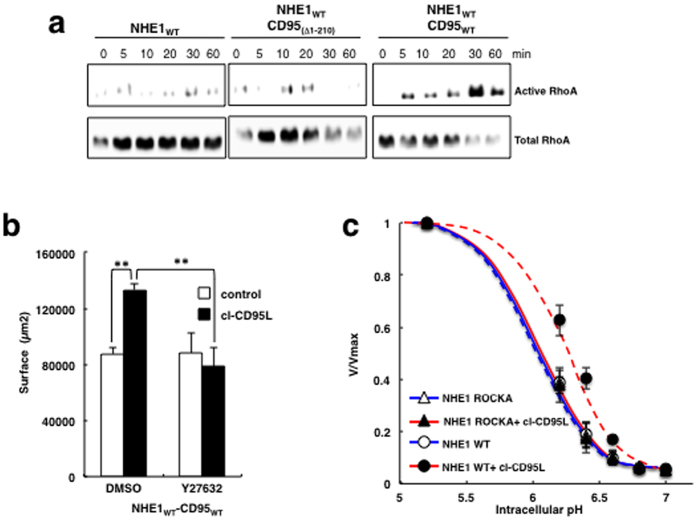
CD95-mediate RhoA stimulation and activates NHE1. (**a**) cl-CD95L activates the RhoA signaling pathway: Cells treated with 100 ng/ml of cl-CD95L for the indicated times were lysed and active RhoA was pulled down using GST-agarose Rhotekin Rho-binding domain. Active and total RhoA were revealed by Western blotting. Data are representative of three independent experiments. (**b**) Cell migration of PS120-CD95-NHE1 wild type was assessed by wound healing in presence or absence of 100 ng/ml of cl-CD95L. Cells were pre-incubated in presence or absence (noted DMSO) of the ROCK1 inhibitor Y27632 (20 μM) for 1 hour. Histogram bars correspond to the surface covered after 24 hours. Error bars are SEMs. p < 0.05 (*), p < 0.01 (**). (**c**) The dose response of the PS120-CD95-NHE1 ROCKA mutant (full lines, ▴) for intracellular protons was measured by Lithium uptake at clamped intracellular pH values in the presence (red line) or absence of cl-CD95L (100 ng/ml) (blue line) as described in Fig. 1d and compared to that the wild type of NHE1 response, (PS120-CD95-NHE1 WT dotted lines, ⦁ red and blue lines). Initial rates of NHE1 are represented normalized values to the maximal uptake (V/Vmax). Data are representative for at least three independent experiments. Error bars are SEM.

The Rho GTPase family plays pivotal roles in regulating the reorganization of the actin cytoskeleton, by activating effector proteins such as p160ROCK, which itself promotes the generation of the actomyosin contractile force^27^. P160ROCK can be activated upon CD95 stimulation, triggering CD95 capping and stimulating the apoptotic signaling pathway^28^,^29^,^30^. p160ROCK has also been shown to activate NHE1 at threonine 653 31, in close vicinity to the Akt phosphorylation site at serine 648. Using a RhoA pull-down assay, we observed that in the presence of cl-CD95L, PS120-NHE1-CD95 cells underwent Rho activation (Fig. 3a), whereas cells devoid of CD95 or expressing a DD-deficient CD95 did not (Fig. 3a, see Materials and methods). In addition, pre-incubation of the cells with the selective p160ROCK inhibitor Y-27632 abolished the CD95-mediated cell motility (Figs 3b and S3C).

**Figure 4:**
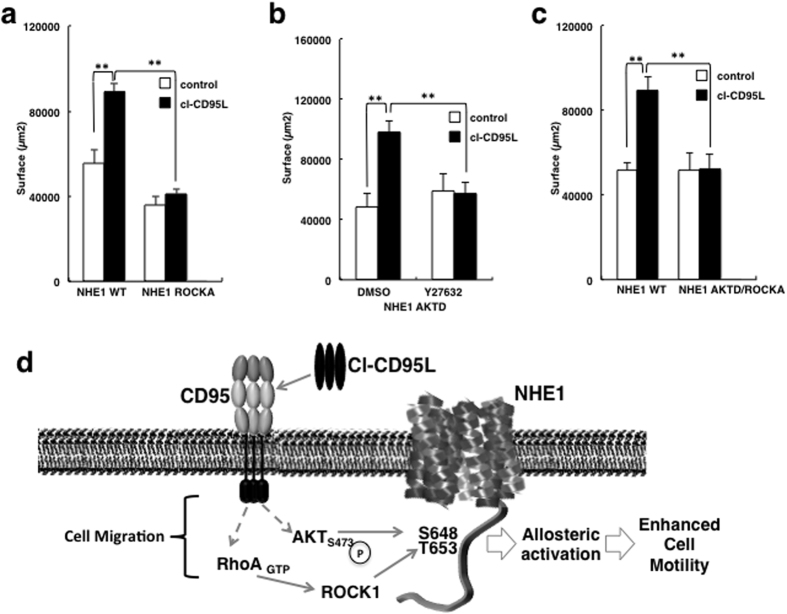
Akt and RhoA pathways cooperate to activate NHE1 and stimulate migration in presence of cl-CD95L. Cell migration of PS120-CD95 cells stably expressing wild type or ROCKA NHE1 mutants was assessed by wound healing assay in the presence or absence of cl-CD95 (100 ng/ml). Histogram bars correspond to the surface covered after 24 hours. Error bars are SEMs. p < 0.05 (*), p < 0.01 (**). (**b**) PS120-CD95 cells stably expressing the AKTD mutant were pre-treated with or without a non-cytotoxic dose of the ROCK1 inhibitor Y27632 (20 μM) for 1 h and then incubated for 24 h in the presence or absence of cl-CD95L (100 ng/ml). Migration was measured by wound healing assay. Histogram bars: surface area covered after 24 h. Error bars are the SEM. (**c**) Cell migration of PS120-CD95 cells stably expressing the AKTD-ROCKA NHE1 double mutants was measured by wound healing assay and compared to that of WT. Histogram bars correspond to the surface covered after 24 hours. Error bars are SEMs. p < 0.05 (*), p < 0.01 (**). (**d**) Summary of NHE1 activation by the non-conventional CD95 engagement.

We then engineered the T653A NHE1 mutant (ROCKA mutant), which cannot be phosphorylated by p160ROCK. ROCKA was fully active and could be stimulated by serum (Fig. S3D), showing that this mutation destroyed neither Na^+/^H^+^ exchange activity nor its regulation. However, in CD95-PS120 cells transfected with this mutant, cl-CD95L was unable to enhance the response of the mutated exchanger to intracellular protons (Fig. 3c) and failed to accelerate cell motility (Fig. 4a). Similarly, both the p160ROCK inhibitor Y-27632 and the ROCKA mutation were sufficient by themselves to abolish the ability of the aforementioned phosphomimetic AKTD mutant to activate NHE1 in response to cl-CD95L (Fig. 4b,c). Together, these results strongly support the notion that cl-CD95L activates NHE1 by a two-step mechanism that requires the activity of both Akt and p160ROCK (Fig. 4d).

### CD95-mediated NHE1 activation in triple negative breast cancer cells stimulates cell migration

Because experiments had been performed in a fibroblast cell system in which the CD95 and NHE1 pathways had been reconstituted by stable transfection, we next evaluated the role of NHE1 in breast tumor cells exposed to cl-CD95L. For this purpose, we first analyzed whether cl-CD95L was able to stimulate Na^+^/H^+^ exchange in BT549 triple negative breast cancer cells (TNBC). As shown in Fig. S4A, cl-CD95L enhanced a reproducible activation of Na^+^/H^+^ exchange by intracellular pH in these breast cancer cells. Second, we evaluated the migratory phenotype of the same BT549 triple negative cells exposed to cl-CD95L in presence or absence of a NHE1 inhibitor (cariporide). We could observe that the cleaved ligand enhanced migration both in Boyden and Wound Healing experiments and that this effect was blocked by 10 μM cariporide (Fig. S4B–D). These data strongly support that cl-CD95L can stimulate NHE1 in TNBC cells to promote cell migration.

To our knowledge, this is the first study demonstrating that cl-CD95 activates the Na^+^/H^+^ exchanger NHE1, showing that it acts through an Akt and ROCK-dependent mechanism and that this in turn contributes to a promigratory signaling pathway. While the promigratory effect of the cleaved CD95 ligand and the role of NHE1 in cell migration had been independently documented in previous studies, the connection between these two events had never been reported. In a different context, in ischemic conditions, NHE1 and CD95 have been shown to interact via the nucleocytoplasmic protein Daxx, which inhibits the Akt1 pathway and enhances CD95-mediated cell death^32^. The present work reveals a completely different, non-apoptotic coupling between NHE1 and CD95 in normoxic conditions. The C-terminal region of NHE1 contains multiple phosphorylation sites (for review, see^24^) and integrates multiple cellular signals to modulate the response of the transporter to intracellular protons. The fact that disrupting the Akt and ROCK phosphorylation sites suppresses cl-CD95 signaling while leaving the global serum response unaffected shows that a combination of key phosphorylation events can convey a very specific signal on NHE1. Consistent with this study, a recent report showed that another dual combination of phosphorylations activates NHE1 to enhance migration, in response to lysophosphatidic acid^33^.

Solving the crystal structure of CD95 revealed that CD95L binding triggers a primary conformational modification of the DD within the receptor, consisting of a shift of helix 6 and its fusion with helix 5 34. This modification promotes both the self-association of the receptor and the recruitment of the adaptor protein FADD. However, this shift was only observed in an acidic context (pH 4) and not in a more neutral pH (pH 6.2)^35^. It is tempting to propose that low pH favors a DD structure that promotes CD95 self-association and FADD binding^34^, leading to cell death^36^. By contrast, activation of NHE1 may prevent the fusion of helix 5 and 6 – perhaps by maintaining locally a higher pH – and thereby counteract FADD recruitment and the execution of the apoptotic signal.

In view of our recent study showing that serum levels of cl-CD95L are a strong prognostic marker of metastatic dissemination in women affected by triple negative breast cancer^7^, and our finding demonstrating that an interplay exists between CD95 and NHE1 pathways to enhance migration of BT549 cells, we can now predict that the activation of NHE1 by cl-CD95L in these patients may contribute to a negative clinical outcome. Accordingly, the results presented in this study raise the possibility that existing NHE1 inhibitors, which have been developed for the management of hypertension and heart failure, may represent new and attractive therapeutic candidates to reduce metastasis in such breast cancers showing high concentrations of serum CD95L, Such molecules might also be of upmost interest for fighting chronic inflammatory diseases in which high levels of cl-CD95 are also detected in patients.

## Methods

### Antibodies and reagents

Anti-human CD95 mAb (DX2) was from BD Biosciences, Anti-Akt, anti-phosphoS473 Akt (Akt-^P473^) were from Cell Signaling Technology. Anti-human NHE1 was from Millipore. The metalloprotease-cleaved CD95L (CD95L) and its multi-aggregated counterpart (Ig-CD95L) were generated in our laboratory, as described previously^3^.

### Cell Culture and Transfection

PS120 fibroblasts^18^ were grown in DMEM with 50 μg/mL streptomycin, 50 unit/mL penicillin, and 7.5% fetal calf serum at 37 °C in a humidified atmosphere of 5% CO_2_ and 95% air. Transfections were performed using Lipofectamin 2000 as described by the manufacturer. Cell populations stably expressing either wild-type or mutant NHEs were selected using 10 μg/ml puromycin for 3 weeks.

### Plasmids construction

CD95 mutant and WT cDNAs have been inserted in the pcDNA3 vector. All NHE1 WT and mutant cDNAs have been inserted in a pECE-NHE1-Ires-Puro polycistronic vector derived from the pECE-NHE1-IresNeo described in^21^.

### Site-directed mutagenesis

Site-directed mutagenesis was performed using Quickchange Site-Directed Mutagenesis kit (Stratagene). Each mutant cDNA was entirely resequenced before and after stable transfection.

### Site-directed mutagenesis primers (sense)

Akt phosphorylation site:

S648A: CAG CGG CTG CGG **GCC** TAC AAC AGA CAC

S648D: CAG CGG CTG CGG **GAC** TAC AAC AGA CAC

ROCK Phosphorylation site:

T653A: TAC AAC AGA CAC **GCG** CTG GTG GCA GAC

T653E: TAC AAC AGA CAC **GAG** CTG GTG GCA GAC

### Immunoblot analysis

Unless stated, proteins prepared from PS120 cells transfected with WT and mutant NHE-1 or CD95 were run on 7.5% acrylamide mini gels (Biorad). Immunoblots were revealed using commercial monoclonal antibodies against NHE1, Akt, phospho Akt and RhoA.

### *In vitro* motility assays

10^5^ cells were added to the top chamber of Boyden chambers (Millipore, Molsheim, France) containing 8 μm pore membranes. The bottom chamber was filled with medium containing 1% serum in the presence or absence of cl-CD95L (100 ng/ml). After 16 hours, cells were fixed with methanol and stained with Giemsa, for visualization. For quantification, invading cells were then lysed and absorbance was measured at 560 nm.

## Wound healing assays

Confluent cells were incubated in 0.5% FCS for 3 hours. We used a home-made plastic guide system to generate straight scratches of similar size in the monolayer. Medium containing 100 ng/ml of cl-CD95 ligand was added and images were acquired for 24 hours using phase contrast microscopy (10× objective Axio Observer D1, Zeiss). Wound healing was quantified using ImageJ (Rasband, W.S., ImageJ, U. S. National Institutes of Health, Bethesda, Maryland, USA, http://imagej.nih.gov/ij/, 1997–2012). For each condition, images were acquired at five different positions along the scratch. The graphs are representative of at least 3 independent experiments.

## Measurement of initial rates of NHE-1

PS120 cells seeded on 24-well plates were acidified as described in (21). Measurements were performed by 1-minute incubation in the uptake medium at room temperature supplemented with 3 mmol/L LiCl followed by four rapid rinses in ice-cold PBS as described in^37^ Cells were solubilized in 1 N nitric acid (trace metal grade, Fisher Scientific), and Li^+^ was measured using atomic absorption spectrometry (Zeeman furnace system, Solaar 969, Thermo Optek). NHE-1 initial rates were calculated as the cariporide (10 μmol/L)–sensitive Li^+^ accumulation per well divided by protein quantities and normalized to maximal values.

## Calcium Video Imaging

Cells were loaded with 1 mM Fura2-AM for 30 min in HBSS. After washing, ratiometric imaging of calcium was performed at 37 °C on a motorizedmicroscope (lens x40, AxioObserver Z1, Zeiss) equipped with an EMCCD camera(Evolve, Photometrics) at 340 and 380 nm. The fluorescence ratios (F_ratio_ 340/380) were analyzed using MetaFluor and Metamorph. Each experiment was repeated 3 times with 60 single-cell traces for each experimental condition.

## RhoA activation

Cells were incubated for 16 hours in 0.5% FCS followed by 3 hours in 0% FCS. They were then stimulated by 100 ng/ml cl-CD95L for the indicated durations. Cells were then lysed and active RhoA was precipitated using the RhoA pull down Rhotekin assay (Thermo Scientific) and revealed by Western Blotting according to the manufacturers instructions. Quantification was performed using Image J.

## Data Treatment and statistical analysis

Data were compiled using Microsoft Excel software. Curves are compiled from at least five independent experiments, in which experimental points are at least duplicates. Data are presented as mean values and Standard Error of the mean (error bars). Statistical analysis was performed using one way ANOVA (SigmaStat 3.01a software, Systat Inc) when testing more than two conditions. Differences were considered as significant when p < 0.05 (*), p < 0.01 (**) and p < 0.001 (***).

## Additional Information

**How to cite this article**: Monet, M. *et al*. The cleaved FAS ligand activates the Na^+^/H^+^ exchanger NHE1 through Akt/ROCK1 to stimulate cell motility. *Sci. Rep*. **6**, 28008; doi: 10.1038/srep28008 (2016).

## Supplementary Material

Supplementary Information
