# Supplementary material for: The cleaved FAS ligand activates the Na+/H+ exchanger NHE1 through Akt/ROCK1 to stimulate cell motility
Source: Sci Rep. 2016 Jun 15;6:28008. doi: 10.1038/srep28008 (PMC4908414; doi:10.1038/srep28008)
Supplement: Supplementary Information [file srep28008-s1.pdf]

Title: The cleaved FAS ligand activates the Na^+/H^+ exchanger NHE1 through Akt/ROCK1 to stimulate cell motility.

Authors : Monet Michael[1§], Poët Mallorie[1], Tauzin Sébastien[2,#], Fouqué Amélie[3], Cophignon Auréa[1], Lagadic-Gossmann Dominique[2], Vacher Pierre[4,5], Legembre Patrick[3,6*] and Counillon Laurent[1,6*]

SUPPLEMENTARY FIGURE 1

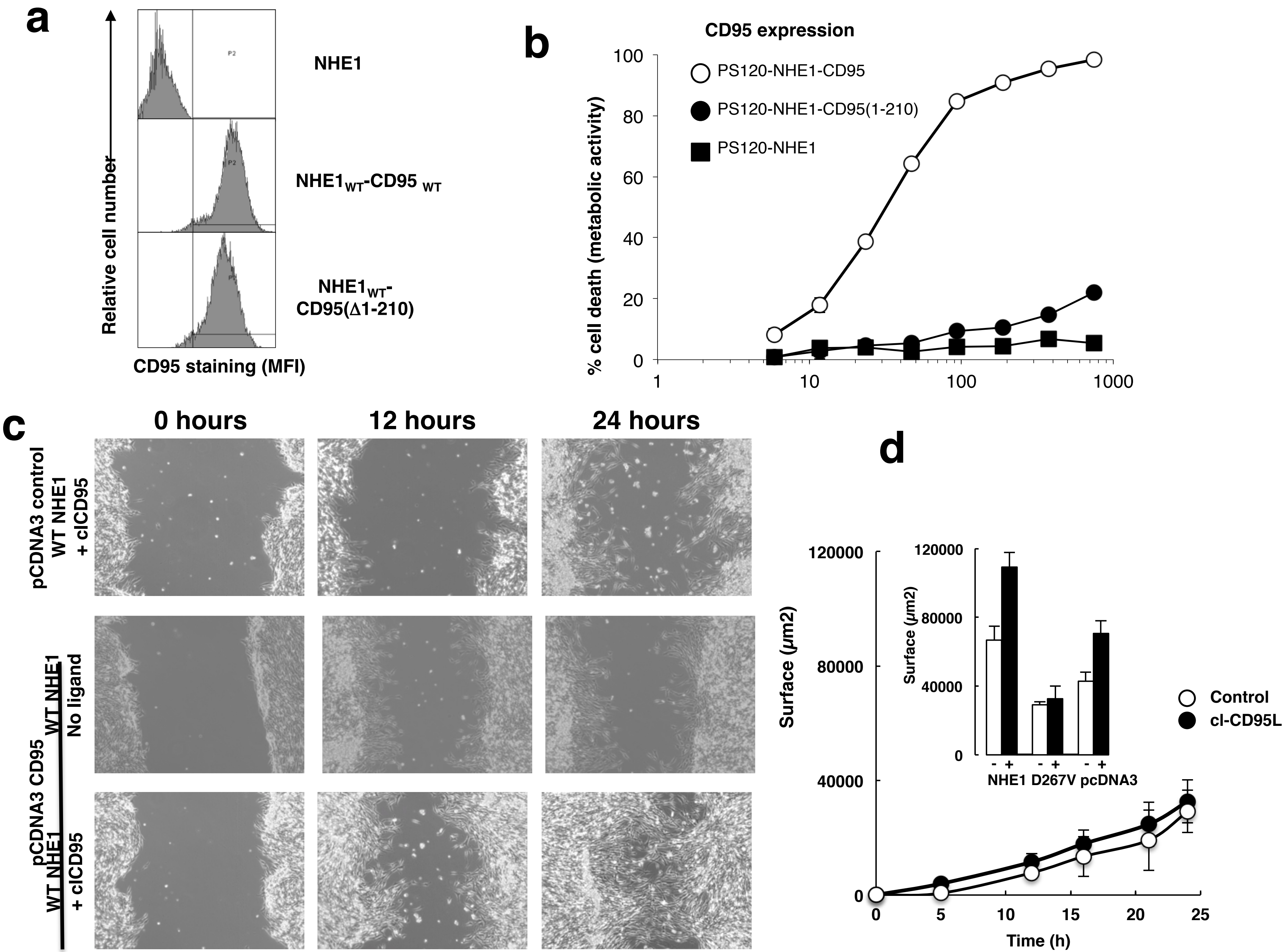

SUPPLEMENTARY FIGURE 2

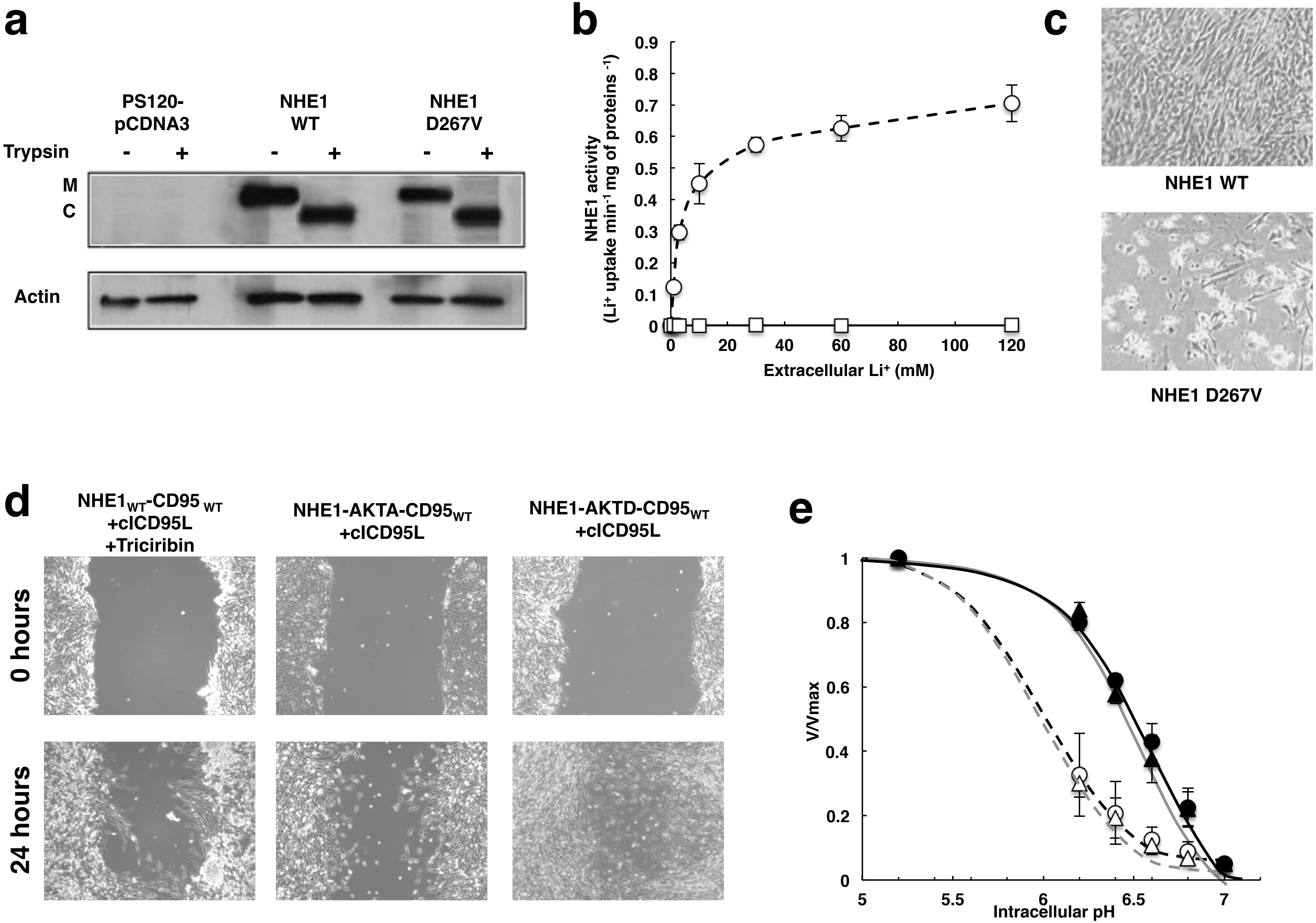

SUPPLEMENTARY FIGURE 3

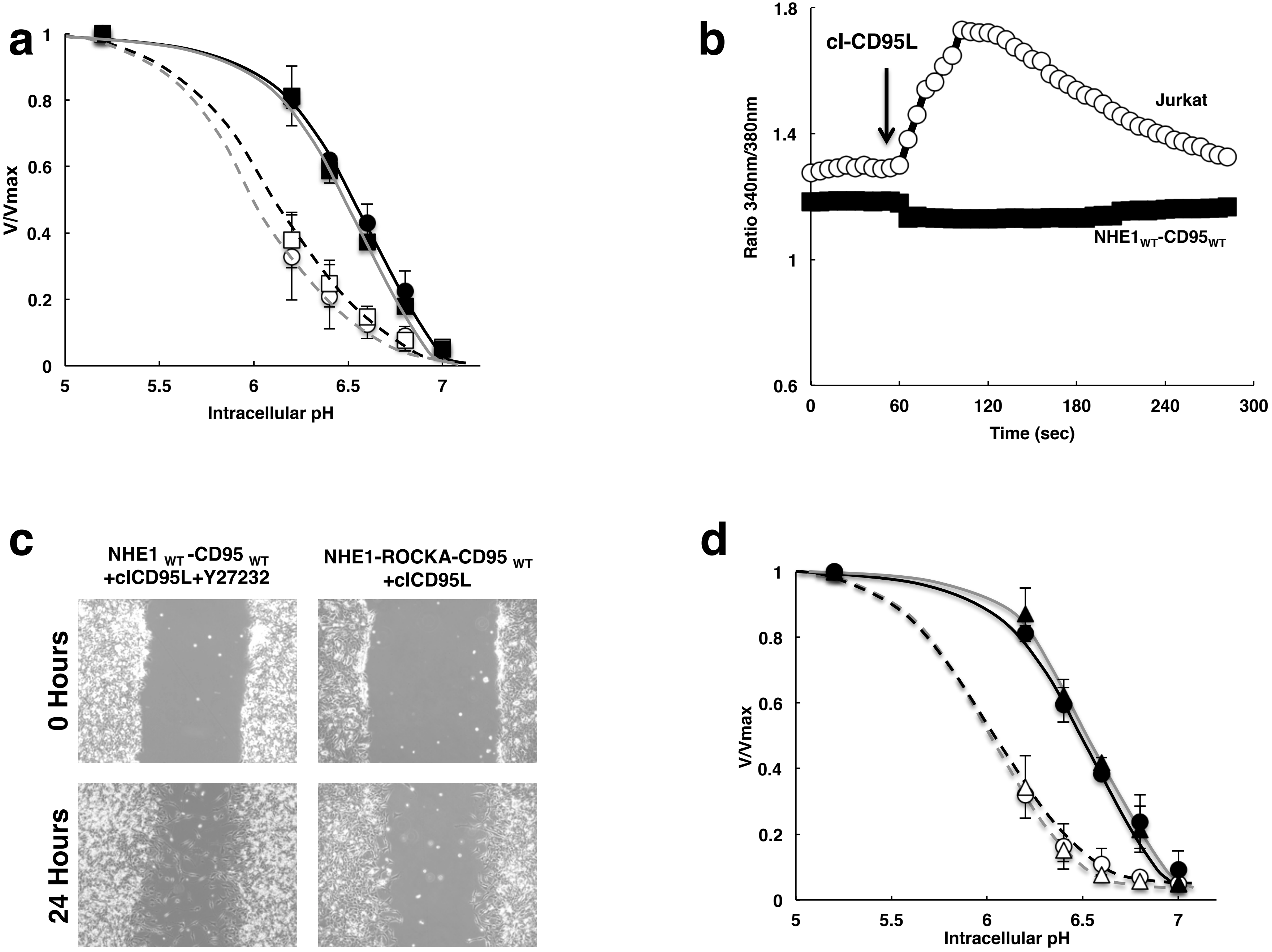

SUPPLEMENTARY FIGURE 4

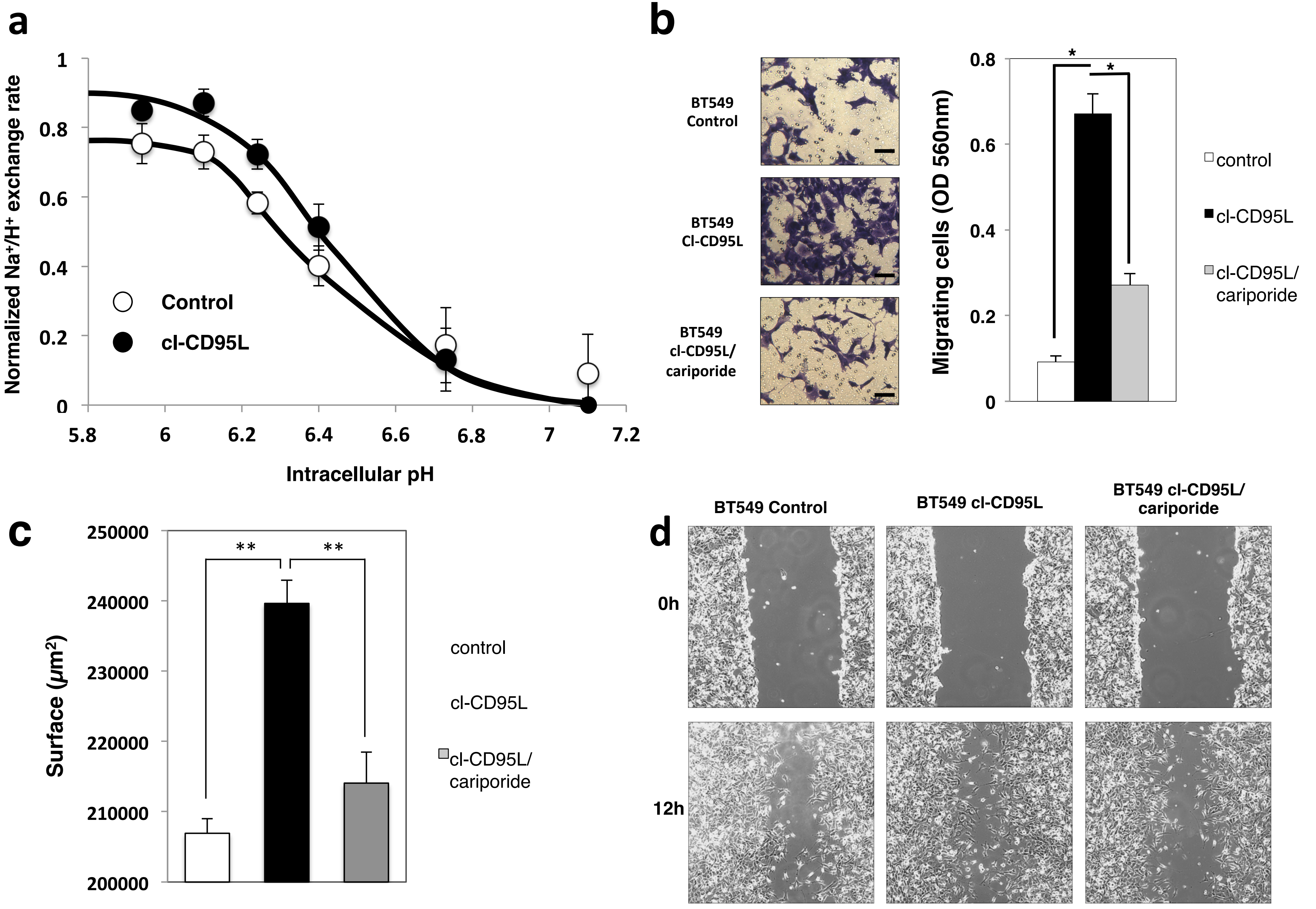

Legends to Supplementary Figures

Figure S1. Effect of CD95 and NHE1 expression on cell migration

A. Expression levels of CD95 in PS120-NHE1 cells were evaluated by flow cytometry. Indicated cells were stained with anti-CD95 mAb (clone DX2) and antibody binding was revealed with a FITC-conjugated secondary antibody. Cell staining was analyzed using a FACSCalibur (BD Bioscience). The mean of fluorescence is correlated to the amount of plasma membrane CD95.

B. Expression of CD95 in PS120-NHE1 cells restores the CD95-mediated apoptotic signal. Indicated cells were incubated for 24 hours with Ig-CD95L and cell viability was assessed using the 3-[4,5-dimethylthiazol-2-yl]-2,5-diphenyltetrazolium bromide (MTT) viability assay.

C. Cell migration of PS120-NHE1 (pCDNA$_3$ Control) and PS120-NHE1-CD95 (pCDNA$_3$CD95) cells was analyzed by wound healing assays in presence or absence of cl-CD95L for 0, 12 and 24 hours. Straight scratches were performed on confluent monolayers and the cells were next exposed to 100 ng/ml cl-CD95L for indicated times. At least five pictures were taken along each scratch and experiments were repeated 3 times each.

D. Migration of PS120-CD95 cells stably expressing the D267V mutant was measured by wound healing and quantified as in C. Inset: Histogram bars correspond to the surface covered after 24 hours. Error bars are SEM. $p < 0.05$ (*), $p < 0.01$ (**).

Figure S2. Expression of the NHE1 D267V and AKTA Mutants

A. PS120 fibroblasts transfected either with empty vector pCDNA$_3$, WT or NHE1 mutant (D267V) were submitted to external trypsin treatment at a final concentration of 0.5 mg/mL for 60 s at room temperature. Trypsin was stopped by two rinses with ice-cold PBS supplemented with 5% BSA. Crude membranes were then prepared. The different forms of NHE-1 (M for mature, C for cleaved,) were separated by SDS-PAGE and visualized by Western blotting.

B. The activities of the WT NHE1 and D267V mutants were measured by initial rates of Li^+ uptake as described in (Milosavljevic et al., 2010 Cancer Res. **70**, 7514-22). WT NHE1: dotted line, O ; D267V mutant : full line, □.

C. Evaluation of cell survival following an acute intracellular acidification (1h, pH 5.2, ammonium prepulse technique) for the PS120 cells stably expressing the WT NHE1 (top panel) or its D267V counterpart (lower panel).

D. Cell migration of PS120-CD95 cells expressing wild type, AKTA or AKTD NHE-1 constructs was evaluated by wound healing assay. Experiments were performed in presence of cl-CD95L (100ng/ml) and of Triciribin, when indicated, for 24 hours. At least five pictures were taken along each scratch and experiments were repeated 3 times each. The quantification is presented in the main figures.

E. The dose response of the AKTA mutant (▲) to intracellular protons was measured in the presence (full lines, full symbols) or absence of 20% FCS (dotted lines, empty symbols) as described in Fig. 2 and compared to that the wild type of NHE1 response (●).

Figure S3. The NHE1 AKTA, AKTD and ROCKA Mutants.

A. The dose response of the AKTD mutant (■) to intracellular protons was measured in the presence (full lines, full symbols) or absence of 20% FCS (dotted lines, empty symbols) as described in Fig. 2 and compared to that the wild type of NHE1 response (●).

B. Jurkat T-cells (❍) and PS120-CD95-NHE1 cells (■) were loaded with 1 mM Fura-2AM for 30 minutes at room temperature. Cells were transferred at 37°C with 2 mM

extracellular calcium and then treated with 100ng/ml of cl-CD95L (black arrow). Values were recorded every 6 s.

C. Cell migration of PS120-CD95-NHE1 cells pre-treated with or without the ROCK1 inhibitor Y27232 (20µM) was evaluated by wound healing assay in presence of cl-CD95L (100 ng/ml) for 24 hours. Similarly, cell migration of PS120-CD95 cells expressing ROCKA mutant was compared to its wild type counterpart. At least five pictures were taken along each scratch and experiments were repeated 3 times each.

D. The dose response of the ROCKA mutant (▲) to intracellular protons was measured in the presence (full lines, full symbols) or absence of 20% FCS (dotted lines, empty symbols) as described in Fig. 2 and compared to that the wild type of NHE1 response (●)

Figure S4. cl-CD95 and NHE1 stimulate motility of Triple Negative Breast Cancer Cells

A. The dose response of the BT549 cells for intracellular protons was measured by Lithium uptake at different intracellular pH values in the presence (●) or absence (❍) of cl-CD95L (100 ng/ml) as described in materials and methods. BTR49 cells were acidified by different times of incubation in 50mM NH_4Cl loading solution followed by a rapid rinse in Choline-Chloride solution as described in [37]. The pH values reached by the different incubations times in the loading solution were measured by BCECF AM (1µM) fluorescence using nigericin calibration in a Synergy HT Biotek device through a combination of two excitation band pass filters centered at 485 nm and 460 nm Initial rates of Na^+/H^+ exchange have been normalized to the maximal uptake (V/Vmax) in the stimulated conditions. Data are representative for at least three independent experiments. Error bars are SEM.

B. **Left panel.** The TNBC cell line BT549 was pre-incubated for 1 h in the presence or absence of cariporide (10µM) and then treated or untreated with cl-CD95L (100ng/ml) for 24 hrs. Cell migration was evaluated by Boyden chamber assay. Migrating cells were fixed and stained by Giemsa. For each experiment, five images of random fields were acquired. Bars=50µm. **Right panel.** To quantify cell migration, migrating cells were lysed and

absorbance was measured at a wavelength of 560 nm. Values represent the means ±SD of three independently performed experiments. (*$P < 0.05$; Mann-Whitney U-test).

C. Cell migration of BT549 cells was analyzed by wound healing assays in presence or absence of cl-CD95L for 0, 12 and 24 hours. Straight scratches were performed on confluent monolayers and the cells were next exposed to 100 ng/ml cl-CD95L and/or 10μM cariporide for indicated times. At least five pictures were taken along each scratch and experiments were repeated 5 times each. Histogram bars correspond to the surface covered after 24 hours. Error bars are SEMs. $p < 0.01$ (**).

D. Illustrative Images corresponding to figure S4.C
